# Rational use of chest ultrasound to confront COVID-19

**DOI:** 10.1590/0100-3984.2020.53.5e3

**Published:** 2020

**Authors:** Miguel José Francisco Neto, Marcos Roberto Gomes de Queiroz

**Affiliations:** 1 Faculdade Israelita de Ciências da Saúde Albert Einstein (FICSAE) - Hospital Albert Einstein, São Paulo, SP, Faculdade de Medicina da Universidade de São Paulo (FMUSP), São Paulo, SP, Brasil. E-mail: mjfneto@uol.com.br.; 2 Faculdade Israelita de Ciências da Saúde Albert Einstein (FICSAE) - Hospital Albert Einstein, São Paulo, SP, Brasil. E-mail: queiroz.radiologia@gmail.com.

There are records of chest ultrasound since the beginning of its use as a diagnostic method, therefore, the mystification and undervaluation of its use is a big mistake^(1)^. Since the 1980s, many publications have demonstrated the importance of this method in chest exams, opening several use possibilities^(2-4)^.

Ultrasonography was increasingly used during the 2000s with the advent of technology miniaturization, which brought high quality images, understanding of the physical bases of subpleural acoustic phenomena and excursion and pleural echo as an essential basis for chest exams, the exact physical explanation for A, B, and C lines, and the routine imaging recording in films represented by point of care ultrasonography^(5-7)^. Urgency protocols are examples of the importance and dimension of the method. The auscultation instrument, created by Laennec, which completed 200 years in 2016, now has a successor in the figure of miniaturized ultrasound^(8,9)^.

The current on-going pandemic caused by the SARS-CoV-2 virus, responsible for the coronavirus disease 2019 (COVID-19) originated in Wuhan, China, in December 2019, has reached global proportions, affecting millions of people in all continents with devastating effects. The clinical presentation of this infection has a broad spectrum and includes pulmonary presentations that range from mild to severe, with rapid and fulminant progression.

The first reports of pulmonary presentations, analyzed by computed tomography (CT) in this disease, show peripheral ground-glass opacities, predominating in the posterior segments of the lung, centrilobular consolidation, and mosaic paving pattern, which can be examined with ultrasound, due to the peripheral location of the lesions^(9,10)^.

Chest radiography is limited to evaluate COVID-19, since it has no adequate sensitivity to detect the ground-glass pattern. CT plays a central role in this pandemic^(10)^. In a multinational consensus, the Fleischner Society^(11)^ discussed the role of various imaging methods to confront the COVID-19 pandemic, focusing on the use of chest radiography and CT.

Ultrasound examination is not part of the most recent protocol of the Brazilian Ministry of Health, which guides to a syndromic diagnosis by clinical history, physical examination, and laboratory confirmation. However, the indications for ultrasound use are structured, and a recent consensus document published by the Brazilian College of Radiology and Diagnostic Imaging details its use^(12)^.

Ultrasound is indicated in some special situations: a) severe involvement of the lower respiratory tract when CT is not available; b) patients progressing with acute clinical deterioration; c) patients who need central venous access; d) difficult intubation cases; and e) patients who need gastric content evaluation to prevent aspiration. The associated use of ultrasound and structured personal protective equipment clearly stands out in emergency care situations, specifically to answer questions such as: a) what is the degree of pulmonary involvement; b) how is the right ventricle preload (diameter/oscillation of the inferior vena cava); c) what is the cause of shock (if any); and d) is the patient ventilating (selective evaluation, atelectasis, pneumothorax)^(12)^. The possibility of being performed in bed and in advanced units, the low cost, the absence of ionizing radiation, and the use of scoring system for the findings, systematizing it analysis in increasing complexity degrees, validate the use of the ultrasound method.

Soldati et al.^(13)^ proposed a scoring scale with classification by field, dividing the rib cage into sectors, and its use as a structured report allows the evaluation of pulmonary presentations, providing not only an early diagnosis, but also the evaluation of disease progression and response to treatment in bed. The studies that use this score can provide structured data. Volpicelli et al.^(14)^ highlights that the disease odds ratio can be graded by categories of pulmonary ultrasound findings. These data can also facilitate studies on a larger scale. Ultrasound exams evaluate complications such as pleural effusion, pneumothorax, venous thrombosis, and abdominal pain.

In the previous issue of the Radiologia Brasileira, the text “Pulmonary ultrasound: an additional tool in COVID-19”^(15)^ was published as a special article on the use of chest ultrasound in COVID-19, presenting the technique, normal findings, disinfection, main findings, and limitations as a screening method. Chest ultrasound is an additional tool in emergency care. Studies on image fusion with CT and microbubble use represent a field of research possibilities^(16,17)^.

The use of imaging methods to diagnose and control treatment in COVID-19 illustrates the central role of imagology in medicine. The special article by Oliveira et al.^(15)^ improves the knowledge of the several ultrasound uses in the fight against COVID-19 .This method, as a medical act, should be thought of as a foundation by the general radiologist.

## References

[1] Yang PC, Luh KT, Chang DB (1992). Value of sonography in determining the nature of pleural effusion: analysis of 320 cases. AJR Am J Roentgenol.

[2] Francisco Neto MJ, Rahal Junior A, Vieira FAC (2016). Advances in lung ultrasound. Einstein (São Paulo).

[3] Lichtenstein D, Goldstein I, Mourgeon E (2004). Comparative diagnostic performances of auscultation, chest radiography, and lung ultrasonography in acute respiratory distress syndrome. Anesthesiology.

[4] Wongwaisayawan S, Suwannanon R, Sawatmongkorngul S (2016). Emergency thoracic US: the essentials. Radiographics.

[5] Lichtenstein DA, Mezière GA (2008). Relevance of lung ultrasound in the diagnosis of acute respiratory failure: the BLUE protocol. Chest.

[6] Perera P, Mailhot T, Riley D (2010). The RUSH exam: Rapid Ultrasound in SHock in the evaluation of the critically ill. Emerg Med Clin North Am.

[7] Hernandez C, Shuler K, Hannan H (2008). C.A.U.S.E.: Cardiac Arrest Ultra-sound Exam-a better approach to managing patients in primary non-arrhythmogenic cardiac arrest. Resuscitation.

[8] Moore CL, Copel JA (2011). Point-of-care ultrasonography. N Engl J Med.

[9] Volpicelli G, Elbarbary M, Blaivas M (2012). International evidence-based recommendations for point-of-care lung ultrasound. Intensive Care Med.

[10] Farias LPG, Fonseca EKUN, Strabelli DG (2020). Imaging findings in COVID-19 pneumonia. Clinics (Sao Paulo).

[11] Rubin GD, Ryerson CJ, Haramati LB (2020). The role of chest imaging in patient management during the COVID-19 pandemic: a multinational consensus statement from the Fleischner Society. Radiology.

[12] Giraldi T, Nocera P, Tonelli AC Recomendações para o uso do ultrassom point of care (POCUS) no atendimento inicial da COVID-19.

[13] Soldati G, Smargiassi A, Inchingolo R (2020). Proposal for international standardization of the use of lung ultrasound for patients with COVID-19: a simple, quantitative, reproducible method. J Ultrasound Med.

[14] Volpicelli G, Lamorte A, Villén T (2020). What's new in lung ultrasound during the COVID-19 pandemic. Intensive Care Med.

[15] Oliveira RR, Rodrigues TP, Silva PSD (2020). Lung ultrasound: an additional tool in COVID-19. Radiol Bras.

[16] Soldati G, Giannasi G, Smargiassi A (2020). Contrast-enhanced ultrasound in patients with COVID-19: pneumonia, acute respiratory distress syndrome, or something else?. J Ultrasound Med..

[17] Ewertsen C, Saftoiu A, Gruionu LG (2013). Real-time image fusion involving diagnostic ultrasound. AJR Am J Roentgenol.

